# 在长载体中引入定点突变的方法

**DOI:** 10.3779/j.issn.1009-3419.2014.07.12

**Published:** 2014-07-20

**Authors:** 凡荣 孟, 琛 陈, 海粟 万, 清华 周

**Affiliations:** 300052 天津，天津医科大学总医院，天津市肺癌研究所，天津市肺癌转移与肿瘤微环境实验室 Tianjin Key Laboratory of Lung Cancer Metastasis and Tumor Microenviroment, Tianjin Lung Cancer Institute, Tianjin Medical University General Hospital, Tianjin 300052, China

**Keywords:** 定点突变, 长载体, Type IIs类限制性内切酶, 桥点引物, Site-directed mutagenesis, Large vector, Type IIs endonuclease, Bridge primer

## Abstract

**背景与目的:**

体外基因定点突变是分子生物学实验的常用方法。然而，虽然目前已经报道了多种基因突变的方法，但对于在长的载体序列中引入突变，一般的方法并不太容易实现。

**方法:**

本研究在我们前期报告的基因突变方法的基础上，描述了一种简单易操作的可在长序列中引入定点突变的方法。这个方法的基本实验程序是：①确定待突变区域，合成一对均含有Type IIs类限制性内切酶位点载体引物，并合成一对互补的突变单链；②在突变区域之外的合适位置上，选择一个桥点，并合成一对均含有Type IIs类的限制性内切酶位点的桥点引物；③利用载体引物序列和桥点引物序列做PCR反应，以扩增载体序列中突变区域外的序列；④利用相应的Type IIs类的限制性内切酶，对以上扩增产物进行酶切；⑤将酶切产物和两个突变单链复性成的突变双链连接，形成突变载体，并转化进受体菌作克隆鉴定。

**结果:**

为证明我们所报告的方法的有效性，我们在长的载体中进行了测试，结果显示，不但实验操作简单易行，而且突变效率可达到90%以上。

**结论:**

我们提供了一种有效的在长载体中进行定点突变的方法。

## Abstract (en)

**Background and objective:**

*In vitro* site-directed mutagenesis is a routine technique in molecular biology labs. However, although there are numbers of related methods available, most of these methods are not suitable for introducing mutations into large vectors.

**Methods:**

In this report, we describe a method which is highly effective for this purpose. Our method is based on the other site-directed method we recently reported. The basic protocol of our method is as follows: (1) Synthesize a pair of vector primers based on the sequences around the region to be mutated, each containing a suitable type IIs endonuclease restriction site; meanwhile, synthesize a pair of short complementary oligonucleotides which forms a mutagenic fragment after annealing; (2) Synthesize a pair of bridge primers which can specifically bind to a site in the vector sequence, each containing a suitable type IIs endonuclease restriction site; (3) Perform PCR reactions using these Vector primers and Bridge primers; (4) Digest the PCR products with the corresponding type IIs restriction enzyme; (5) Ligate the digested fragment with the mutagenic fragment to make the desired mutant.

**Results:**

Using this protocol, we have introduced mutations into a vector larger than 9 kb. The results shows that the mutation rates are more that 90%.

**Conclusion:**

Our method provides a useful tool for performing site-directed mutagenesis experiment in large vector.

体外基因定点突变（*in vitro* site-directed mutagenesis）是分子生物学领域一种常用的技术手段^[1-3]^。目前为止，已经报告了多种实现基因定点突变目的的实验方法^[4, 5]^。这些方法虽然各有特点，但大多都是利用突变引物将所需要的突变引入载体序列，也能满足大多数的实验要求。只是在多数情况下，待突变的序列一般都位于7 kb以下的载体上，在这些载体进行定点是容易实现的。然而，对于长的载体序列，许多方法就不适用，其中原因，一是利用突变引物做扩增时，效率较低，二是后续实验中突变效率也很低。尽管有人报告了一些解决这一问题的方法^[6, 7]^，但大多在操作上比较繁琐。

我们最近报告一种不依赖于突变引物的实验方法。这种方法借助一对含有Type IIs类限制性内切酶位点的载体引物^[6-10]^，以及一个含有所需要的突变的短的DNA双链，实验利用载体引物对除突变区域外的载体序列进行扩增，然后，利用相应的Type IIs类的限制性内切酶对扩增产物进行酶切，并与突变双链进行连接以形成突变载体^[11]^。该方法的突变效率接近100%。本文所描述的对长载体序列进行突变的方法，就是在这个已经报告的方法的基础上形成的。本文的实验，在长载体序列上进行了突变，结果表明，我们所提供的在长载体序列进行突变的方法是一个非常有效的手段和工具。

## 1 材料和方法

### 1.1 材料

本文所用的两个载体为本实验室保存质粒，分别为质粒pOCT4-Luc1和pOCT4-Luc2，两个质粒长度均为9, 613 bp，其中质粒pOCT4-Luc2（载体序列见附录）含有一个限制性内切酶*Esp*3I的酶切位点，质粒pOCT4-Luc1中没有这个酶切位点，其余部分序列与pOCT4-Luc2相同。实验中所用材料为限制性内切酶*Es*p3I和*Dpn*I（Fermentas公司）、PCR反应试剂（DR010A, Takara）、T4 DNA Ligase、DNA产物纯化试剂盒（DNA Fragment Purification Kit, DV807A, TaKaRa），DH5a感受态均为大连宝生物有限公司产品，质粒小提试剂盒购自天根生化科技北京有限公司。

### 1.2 方法

#### 1.2.1 引物合成

本文用到突变DNA双链为Mutagenic fragment，所设计的引物为V-primer A, B；B-primer A1, B1；B-primer A2, B2。三对引物中均含有一个Type IIs类的限制性内切酶，*Esp*3I的酶切位点，相应酶切后产生粘性末端便于下一步骤的连接反应。实验中所用的引物由华大基因科技服务有限公司合成，具体序列见表 1。

**1:** 实验所使用的突变引物序列 Oligonucleotides used in the experiments

	Sequence 5'-3'
V-primer A	5' AAGCT CGTCTC TCGCACATATCGAGGTGGACATTAC 3'
V-primer B	5' GGATA CGTCTC TGCGTAGCGCTTCATGGCTTTGTGCA 3'
B-primer A1	5' GGCAT CGTCTC TGTGTAGTGCTGCCATTACCATGAG 3'
B-primer B1	5' CGAAT CGTCTC AACACAATTCTCTTACCGTCATGCCATCCG 3'
B-primer A2	5' AAGCT CGTCTC TACTGTGTTCCATGGTGACTGTAGGTGATGC 3'
B-primer B2	5' GGATA CGTCTC TCAGTGGAATGGTGCCTAAAGCCCTGGTG 3'
Mutagenic fragment A	5' ACGC CCTAGCGCCCGGCATCGCCTCTGCATTGT 3'
Mutagenic fragment B	5' TCGG ACAATGCAGAGGCGATGCCGGGCGCTAGG 3’

#### 1.2.2 突变片段及PCR产物的制备

人工合成的一对互补的突变单链，复性得到突变片段，此反应在PCR仪中完成。PCR反应所用模板为质粒pOCT4-Luc1和pOCT4-Luc2，所用聚合酶为PrimeSTAR HS DNA Polymerase（DR010A, Takara）；引物分别为V-primer A、B-primer A1；V-primer B、B-primer B1；V-primer A、B-primer A2；V-primer B、B-primer B2。PCR反应参数：98 ℃、30 s后进入循环，98 ℃、10 s，62.5 ℃、15 s，72 ℃、6 min，反应28个循环，最后于72 ℃下保持20 min。所得PCR产物保存备用。

#### 1.2.3 模板质粒去除

将所得PCR产物直接转入37 ℃水浴，加入*Dpn*I酶40 U，酶切1 h。目的是去除模板质粒，原因是*Dpn*I能够识别甲基化位点并将其酶切^[12, 13]^，而PCR产物是没有甲基化的，所以*Dpn*I酶能够特异性地切割模板（质粒）而不会影响PCR产物，从而去掉模板留下PCR产物。

#### 1.2.4 连接粘性末端的形成及连接反应

将上一步骤所得产物进行纯化，所用试剂盒为DNA Fragment Purification（DV807A, Takara）。然后用限制性内切酶*Esp*3I进行酶切，形成粘性末端，反应条件为37 ℃水浴2 h。再次进行DNA纯化。最后进行连接反应，反应条件为16 ℃水浴2 h，所用试剂T4 DNA Ligase（Takara）。

#### 1.2.5 化学转化、质粒鉴定及测序

取所得连接产物1 µL，转入100 µL感受态细胞混匀，冰上放置30 min，42 ℃水浴1 min后置于冰上2 min-3 min。涂布于含amp抗生素的LB平板，37 ℃培养过夜后挑单菌落鉴定质粒。提取质粒使用天根质粒小提试剂盒。所得质粒在华大基因科技服务有限公司进行测序。

## 2 结果

### 2.1 在长序列中引入突变的工作流程

最近我们报告了一种不依赖于突变引物的进行基因定点突变的方法。这里所描述的方法，就是在我们前期所报告的方法的基础上形成的^[14, 15]^。如图 1所示意，是在长序列中引入突变的基本流程：①在待突变的区域的两侧，合成一对载体引物V-primer A和V-primer B，这一对载体引物序列中均含有一个合适的Type IIs类的限制性内切酶位点，此外，还合成两个突变单链并复性融合成突变双链；②选择一个桥点（bridge site），桥点的位置，大概是距离两个载体引物结合区的距离比较接近的区域，实验合成一对桥点引物B-primer A和B-primer B，每个桥点引物中也含有一个Type IIs类的限制性内切酶位点；③利用V-primer A和B-primer A配对，V-primer B和B-primer B配对，分别进行PCR反应，对扩增产物进行纯化后，利用相应的Type IIs类的限制性内切酶进行酶切；④将以上酶切产物和突变双链混合并作连接反应以形成突变载体；⑤将连接产物转入受体菌并对所选择的克隆进行鉴定。在这里的实验程序中^[16, 17]^，我们借助了前期报告的方法的简单和效率，又利用一个桥点，将长的载体序列分成两个片段进行扩增，这样既解决了长载体中进行突变的效率问题，也解决了长载体中进行突变的扩增问题。这个实验程序是否可用，将在后续的实验中进行验证。

**1:**
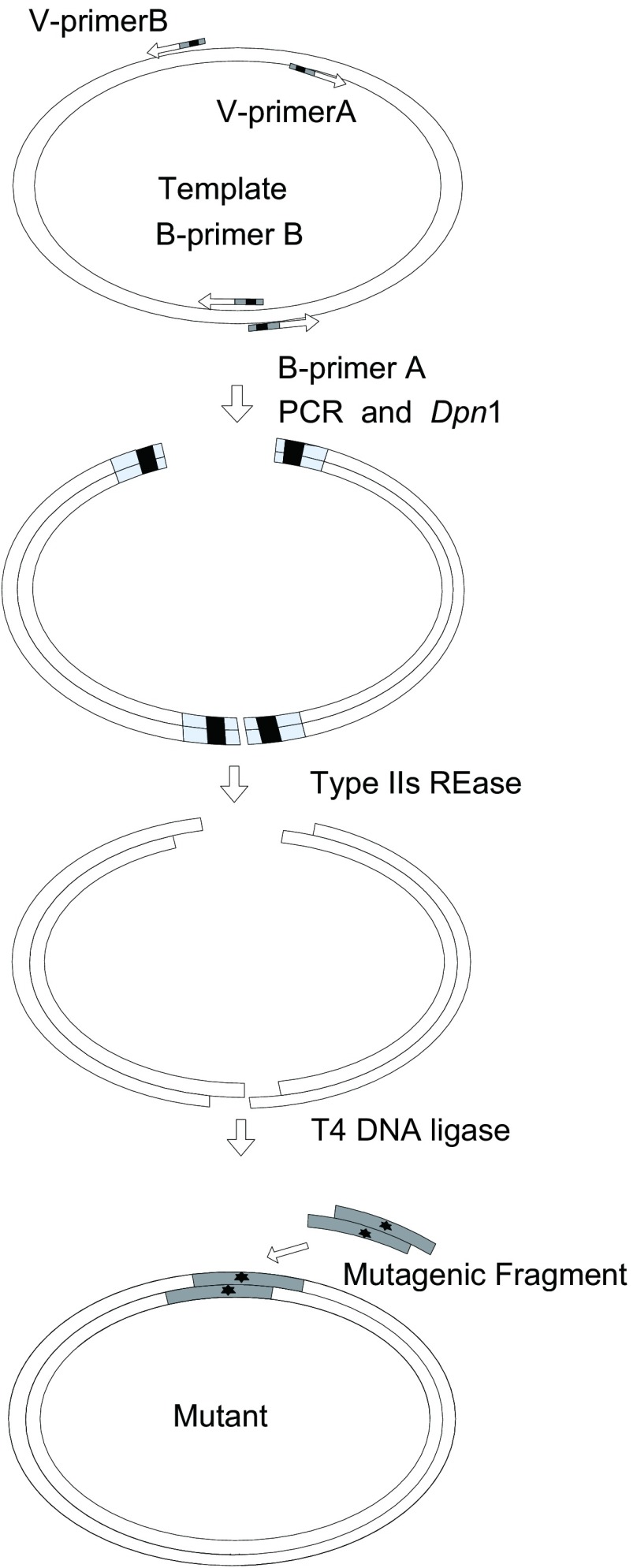
实验流程示意图 Outline the experimental procedure of our method. A pair of vector primers and a short mutagenic fragment are synthesized, each vector primers containing a type IIs class endonuclease site, simultaneously, a bridge site is selected and a pair of bridge primers is also synthesized and each of the bridges primers contains a type IIs class endonuclease site. Two PCR reaction are then performed, each using a vector primer and a bridge primer. The PCR products are next digested with Dpn I and the corresponding type IIs class endonuclease. The digested products are subsequently ligated with the mutagenic fragment to form the mutant.

### 2.2 在长载体中引入复杂定点突变

为验证以上实验程序的有效性，我们选择了实验室已有的最长的载体pOCT4-Luc1和pOCT4-Luc2，该载体的长度为9, 613 bp，远大于常见的7 kb以下的载体。此次实验中使用的突变片段及配对引物见图 2。以质粒pOCT4-Luc1为例，对此实验过程进行阐述，如图 3所示意，实验选择在该载体的报告基因中，引入复杂突变片段Mutagenic fragment，其中包括了置换，删除和插入等。我们合成了一对载体引物，V-primer A和V-primer B，这对引物中均含有一个Type IIs类的限制性内切酶位点，即*Esp*3I位点。实验选择了一个桥点，该桥点位于载体序列中，实验合成了一对桥点引物，B-primerA1和B-primerB1，其中，每个引物中均含有一个*Esp*3I位点。实验进行两个PCR反应，其中一个以V-primer A和B-primer A1为引物，而另一个则以V-primer B和B-primer B1为引物，这样就分别获得两个PCR产物，一个长度为3, 387 bp，另一个长度为6, 188 bp；实验继续借助*Esp*3I对PCR产物进行酶切，所获得的酶切产物和突变片段进行连接，就形成了所需要的突变载体1-AA1BB1。在经过对突变克隆进行筛选和测序鉴定后，被测序的14个克隆中，其中13个克隆含有所需要的突变，突变率达到93%。在质粒pOCT4-Luc1中还合成了另外一对桥点引物来证明方法的可行性，实验进行的两个PCR反应，其中一个以V-primer A和B-primer A2为引物，另一个则以V-primer B和B-primer B2为引物，这样获得的两个PCR片段，一个长度为4, 942 bp，另一个长度为4, 633 bp，进行酶切后与突变双链进行连接，构成突变载体1-AA2BB2，突变率为90%。这个结果充分说明了我们所提供的方法的有效性。图 3具体阐述了整个实验的流程，引物中与模板质粒互补的碱基序列用下划线标出，PCR反应后用限制性内切酶*Esp*3I进行酶切，暴露出粘性末端，与突变片段进行连接反应。突变片段中被突变的碱基用下划线标出。

**2:**
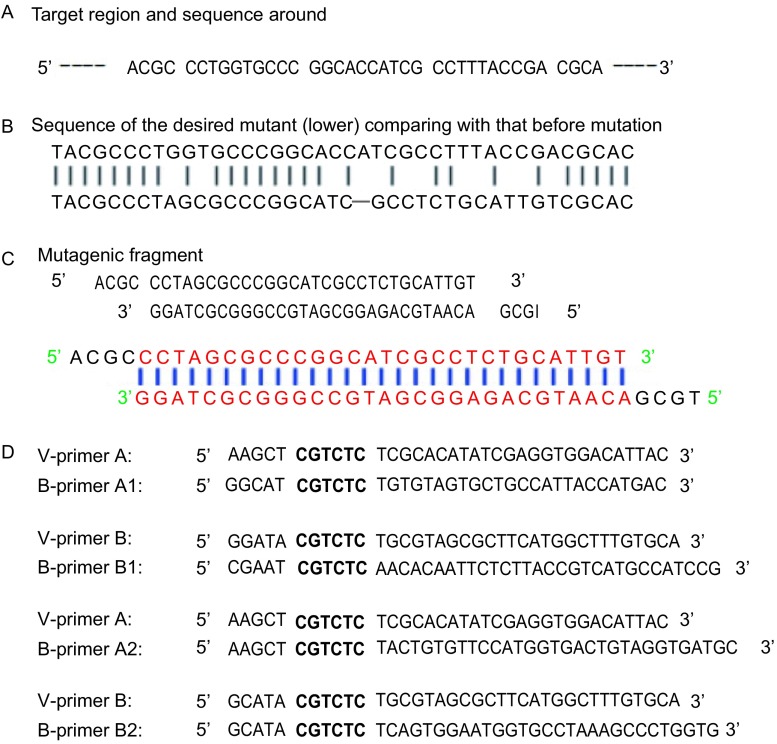
实验中所使用的碱基序列 Oligonucleotides used in the experiments

**3:**
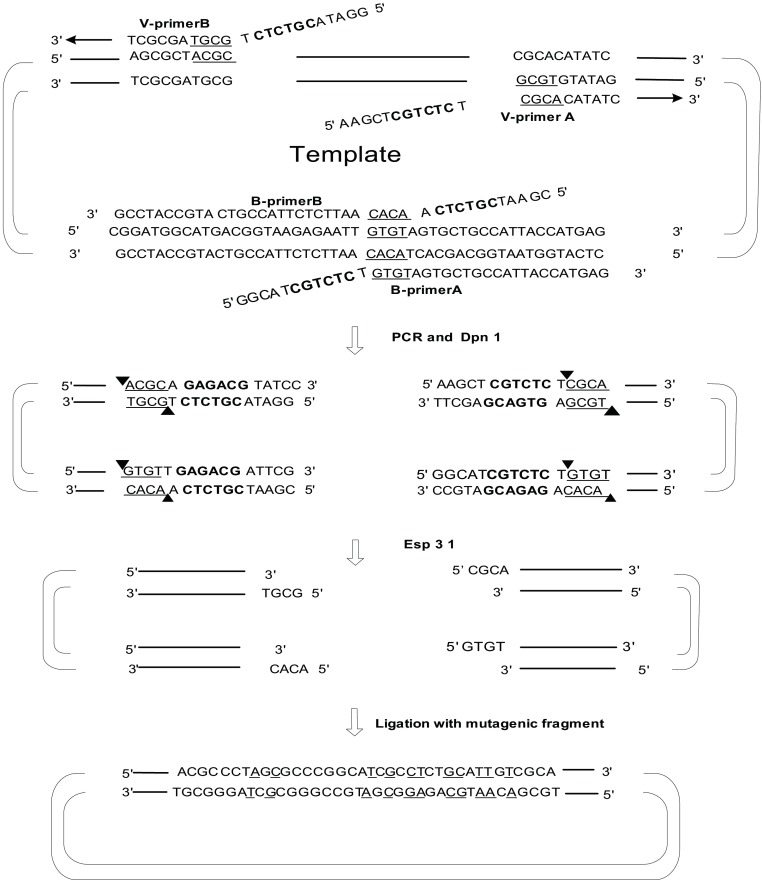
实验步骤。图中*Esp*3I酶切位点用黑体字标出，被突变的碱基用下划线标示。 The experimental procedure. The type IIs restriction enzyme sites, *Esp*3I, are in bold and the mutational sites are underlined.

在长的载体序列上，可能在载体序列内部，含有相应Type IIs类限制性内切酶位点。为证明我们提供的方法对这一类载体的有效性^[18, 19]^，我们选择了另一个载体pOCT4-Luc2，该载体和前面的实验的区别，仅是在载体序列中含有一个*Esp*3I位点，其它部分没有区别。我们利用这个载体，分别以V-primer A、B-primer A1；V-primer B、B-primer B1和V-primer A、B-primer A2；V-primer B、B-primer B2进行了以上所描述的实验，这样在借助*Esp* 3I位点进行酶切时，就多出现一个片段，最后进行连接反应时实际上是四个DNA片段进行连接，得到的突变载体为2-AA1BB1、2-AA2BB2。，实验最后挑取单克隆，并对其中所含有的载体序列进行测序，结果表明，突变载体2-AA1BB1测序的12个克隆中，其中12个克隆含有所需要的突变，突变率达到100%；突变载体2-AA2BB2测序的11个克隆中，其中11个克隆含有所需要的突变，突变率达到100%。这个结果说明，我们所提供的方法，对内部含有Type IIs限制性内切酶位点的序列也有很好的突变效率。图 4A显示了实验中四个突变载体的突变效率，图 4B为突变片段的测序图。

**4:**
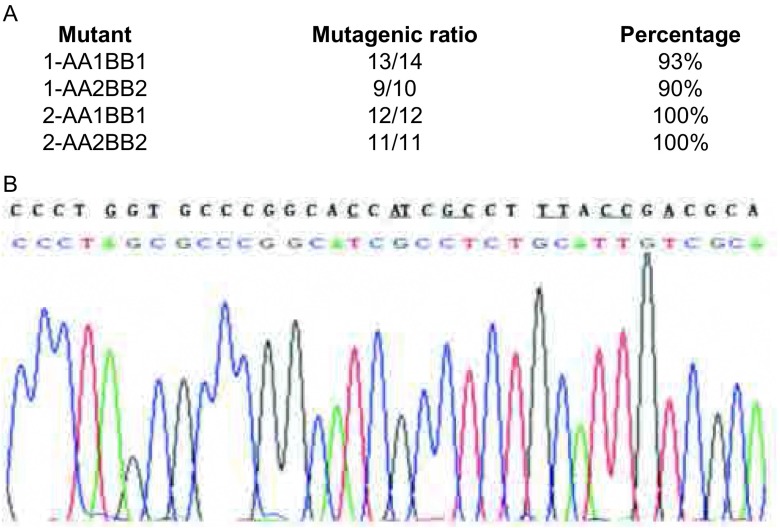
实验结果及测序图。A：突变的成功率；B：实验测序图。 Sequencing results of the mutants. A: Mutational rate of the mutants; B: Sequencing maps of the mutants.

## 3 讨论

我们描述了一种适合在长载体序列中引入定点突变的方法。这种方法是在我们最近报告的依赖于Type IIs类的限制性内切酶和人工化学合成的突变双链的突变方法的基础上形成的。我们的方法利用了最近所报告的方法的高效特点^[20]^，另一方面，又利用在载体序列的合适位置上的桥点，从而将长的载体序列分成几个小于7 kb的短的序列进行扩增。我们在一个超过9 kb载体序列上，成功地引入了复杂突变。另一方面，由于在实验中使用了Type IIs类的限制性内切酶，在长的载体序列的内部，也有可能出现这样的位点，我们对这种情况下的突变也进行了检测，结果也表明，载体序列内部的与所选择的Type IIs类限制性内切酶一致的位点，对实验不构成影响。在我们的实验中，所使用的Type IIs类的限制性内酶位点是*Esp*3I位点，这个位点可以满足大部分的实验要求，不过，如果实验需要，也可以选择其它的类似的限制性内切酶位点^[21, 22]^，例如*Sap*I等。我们所提供的方法，实验操作非常简单。从序列的PCR扩增到将连接产物转入受体菌，一天之内就可以完成。在我们的实验操作中，我们一般会提前一天进行PCR反应，那么，第二天的工作就可以轻松完成。相对与其它类似的方法，我们所提供的方法具有很大的优势。我们所描述的方法，为在长载体序列中进行突变提供了有效的工具。
